# Incorporating Therapeutic Education and Exercise in Migraine Management: A Biobehavioral Approach

**DOI:** 10.3390/jcm13206273

**Published:** 2024-10-21

**Authors:** Roy La Touche, Arão Belitardo de Oliveira, Alba Paris-Alemany, Álvaro Reina-Varona

**Affiliations:** 1 Centro Superior de Estudios Universitarios La Salle, Universidad Autónoma de Madrid, 28023 Madrid, Spain; roylatouche@yahoo.es (R.L.T.); alvaroreina93@gmail.com (Á.R.-V.); 2 Motion in Brains Research Group, Centro Superior de Estudios Universitarios La Salle, Universidad Autónoma de Madrid, Aravaca, 28023 Madrid, Spain; 3 Instituto de Dolor Craneofacial y Neuromusculoesquelético (INDCRAN), 28008 Madrid, Spain; 4 Center for Clinical and Epidemiological Research, Hospital Universitário, Universidade de Sao Paulo, Sao Paulo 05508-220, Brazil; araoliva@gmail.com; 5 Departamento de Radiología, Rehabilitación y Fisioterapia, Facultad de Enfermería, Fisioterapia y Podología, Universidad Complutense de Madrid, 28040 Madrid, Spain; 6 PhD Program in Medicine and Surgery, Doctoral School, Universidad Autónoma de Madrid, 28029 Madrid, Spain

**Keywords:** migraine disorder, therapeutic exercise, biobehavioral education, motivational interviewing, lifestyle recommendations

## Abstract

The main objective was to perform a description of the potential biobehavioral factors that influence disability in patients with migraines and develop a multimodal physiotherapy treatment proposal incorporating therapeutic education and exercise prescription, applying a biobehavioral approach. This manuscript highlights the complex interplay between migraines and physical activity, with many migraine sufferers performing reduced physical activity, even during headache-free intervals. The kinesiophobia present in a significant portion of patients with migraine exacerbates functional disability and compromises quality of life. Psychological elements, especially pain catastrophizing, depression, and self-efficacy, further compound migraine-related disability. Addressing these issues requires a multidisciplinary approach that integrates physical activity and behavioral interventions. We propose a therapeutic education model of motor behavior that emphasizes the enhancement of therapeutic exercise outcomes. This model consists of the four following phases: (1) biobehavioral analysis of movement; (2) goal setting; (3) education about exercise benefits; and (4) movement education. A notable feature is the incorporation of motivational interviewing, a communication strategy that amplifies intrinsic motivation for change. Recent clinical guidelines have advocated for specific exercise modalities to ameliorate migraine symptoms. However, we highlight the importance of a tailored exercise prescription to maximize the benefits of exercise and reduce the possible adverse effects. The integration of exercise with other lifestyle recommendations, such as maintaining consistent sleep patterns and employing stress management techniques, is pivotal for improving outcomes in patients with migraine. Although evidence supports the benefits of these interventions in various painful conditions, further research is needed to establish their efficacy specifically for migraine management.

## 1. Introduction

Migraine is a debilitating neurological disorder [1], affecting over a billion individuals globally. It is frequently characterized by recurrent and taxing headaches, some of which are accompanied by an aura [2,3]. This disorder imposes a significant economic burden worldwide, detrimentally impacting the person with migraine’s quality of life, as well as their family, coworkers, and society at large [2,4,5].

In terms of prevalence, migraines are considered the second most significant contributor to disability on a global scale, and the foremost cause of disability among young women [6]. The profound effect of migraines on quality of life cannot be understated, due to its influence on professional spheres, family, and social relationships, resulting in pronounced emotional decline [7,8].

Migraine pathophysiology is associated with various alterations in both the central and peripheral nervous systems [1]. Neuroimaging and electrophysiological studies have identified both structural and functional aberrations in the brains of patients with migraine [9,10,11,12]. Spread depolarization appears to be the root cause of the aura and can also trigger trigeminal sensory activation, which is the underlying mechanism of headache pain [3]. Theoretical models applied to patients with migraines suggest that augmented and sustained pain perception induces neuroplastic changes in the central nervous system [13]. These changes potentially impact motor behavior and are influenced by contextual and cognitive–emotional factors (fear-avoidance beliefs, feelings of reduced self-efficacy, catastrophic cognition, and increased depressive symptoms) [14,15,16]. It is suggested that maladaptive changes in motor behavior can heighten disability levels and pain perception and can further reduce quality of life [17].

Understanding migraines from a biobehavioral perspective entails acknowledging their biological foundations and their interplay with psychological elements pertinent to patients’ symptomatology. Those with migraines frequently endure psychological distress, including symptoms of depression and anxiety, and they face societal stigma and misunderstanding [7,8,18,19]. Research has shown that psychological elements, such as anxiety, depression, and pain catastrophizing, show a robust relationship with migraine disability [20]. A meta-analysis performed by Waliszewska-Prosól et al. (2024) revealed that the pooled mean value for the MIDAS questionnaire score obtained by migraine patients in different studies was 36.8, with 62.3 for HIT-6 and 58.1 for HDI [21]. A score higher than 21 on the MIDAS questionnaire is considered a severe disability [22], more than 60 on the HIT-6 is regarded as a very severe disability [23], and more than 50% is also interpreted as a severe disability in the HDI questionnaire [24]. Considering the high disability present in these patients, and its association with the mentioned psychological factors, the management of these barriers might be crucial for decreasing the impact of this condition in patients’ lives.

The therapeutic landscape of migraines has been extensively researched and reviewed, covering both pharmacological and non-pharmacological treatments [3,25,26]. Although drugs are the typical treatment, they pose challenges, such as drug interactions, patient response variability, and safety concerns, which are intensified when polytherapy is required to address comorbidities or to achieve a sufficient therapeutic effect [26].

Given the heterogeneity in treatment response, it is important to consider the patients’ perspectives. Qualitative studies delving into the views of those with migraines have highlighted challenges in accessing healthcare, establishing relationships with providers, having aversions to prescribed medications, and a preference for non-drug treatments [25]. They have also highlighted the importance of understanding the social implications and diagnosis, as well as knowing the potential migraine triggers.

Beyond pharmacological approaches, behavioral interventions have emerged as a promising alternative that is backed by robust scientific evidence. Effective patient–healthcare provider communication is essential, recognizing individual factors influencing migraine onset and management [27]. Among behavioral strategies, exercise, notably aerobic, has been pinpointed as a promising preventive treatment, with significant symptomatic improvements having been observed [28]. Such is the impact of exercise on these patients that various scientific societies related to headaches have included exercise as part of their non-pharmacological treatment recommendations [29,30,31]. Moreover, multiple authors have highlighted the efficacy of behavioral interventions, such as relaxation and biofeedback, in migraine prevention and management [27,32,33]. When combined with pharmacotherapy, these techniques have proven particularly advantageous [27].

This manuscript will describe the potential biobehavioral factors influencing disability and other alterations affecting patients with migraine. Furthermore, it will detail a multimodal physiotherapy approach plan grounded in a biobehavioral framework, incorporating therapeutic education and an evidence-based exercise prescription.

## 2. Migraines and Physical Activity

The nexus between physical activity (PA) and migraines has been scrutinized in various scientific contexts. Though findings differ, creating a complex and intricate landscape, PA remains a pivotal topic in therapeutic and diagnostic approaches, particularly from a physiotherapy perspective.

The relationship between physical activity and migraines has been extensively investigated in numerous studies. While intense exercise can act as a trigger for attacks in certain individuals, it has also been shown to play a prophylactic role in reducing the frequency and severity of migraine episodes. In a comprehensive review, Amin et al. (2018) analyzed the evidence from epidemiological, therapeutic, and pathophysiological perspectives, highlighting that potential triggering mechanisms include the release of neuropeptides, such as calcitonin gene-related peptide (CGRP), and alterations in lactate metabolism during physical activity. However, regular exercise may also increase the migraine activation threshold, acting as a protective factor in individuals who engage in it frequently [34].

Regarding prevalence, Koppen and Van Veldhoven (2013) reported that approximately 38% of patients experienced exercise-induced migraines, with neck pain being a more common initial symptom in these episodes compared to their typical migraines [35]. However, Varkey et al. (2017) observed that after subjecting migraine patients to maximal exercise tests, not all experienced attacks, suggesting significant variability in individual susceptibility to exercise triggers [36]. This variability may be influenced by factors such as the baseline frequency of migraine attacks.

On the other hand, the intentional avoidance of exercise is a frequent phenomenon among migraine patients, as noted by Farris et al. (2018, 2019). These studies reveal that avoidance of physical activity, especially high-intensity exercise, is associated with a higher frequency of migraine attacks, suggesting that physical inactivity could contribute to the perpetuation of migraine episodes [37]. Additionally, patients who avoid physical activity tend to believe that exercise will worsen their symptoms, highlighting the need for interventions aimed at modifying these erroneous beliefs and promoting controlled physical activity as part of comprehensive migraine management [38].

Several researchers have evaluated the PA levels of patients with migraine in comparison to asymptomatic participants and those with other headache types. In this context, Rogers et al. (2020) compared PA levels among adolescents with migraines, those experiencing tension-type headaches, and headache-free controls. Those with migraines displayed reduced overall PA compared with those without headaches, not solely during headache episodes but also on days without headaches, suggesting a general reduction in PA for migraine-afflicted individuals [39]. Aligning with these findings, Stronks et al. discerned that patients with migraines, while having heart rates comparable to controls, were notably less physically active in their routine settings. Moreover, their bodily motion during activity was subpar compared with controls. These interictal phenomena of low physical activity reinforce the chronic impact of migraines on patients’ daily lives [40].

Physical inactivity seems to also contribute to migraine onset. As posited by Varkey et al., those with a sedentary lifestyle had a heightened probability of developing non-migraine headaches a decade later. Furthermore, reduced PA was correlated with a surge in both migraine and non-migraine headache prevalence [41]. Reinforcing these findings, recent research has illustrated that inactive individuals have a higher migraine prevalence compared with their active or highly active counterparts. Notably, population-based studies showed that PA levels were inversely associated with depression, anxiety, negative self-perceived health, and analgesic use [42], as well as headache disability [43].

Compelling data have also emerged from the ELSA-Brasil cohort studies [44,45], which pointed to a link between physical inactivity and the frequency of migraine attacks. These findings suggest that adhering to World Health Organization (WHO) PA guidelines could mitigate migraine occurrence, with leisure-time PA being especially significant [44]. Further findings from the same cohort (2022) highlighted that those meeting the WHO’s PA criteria had a decreased migraine occurrence. Moreover, the type and intensity of PA could have distinct roles concerning migraine subtype [45]. In the All of Us study, a prospective study with 6042 participants using wearable devices and analyzed during a 4-year period, each additional increment of 1000 daily steps beyond the sample median resulted in an 8% reduction in the incidence of migraines [46]. The study by Domingues et al. (2011) noted reduced functional disability in a sample of medical students with migraine who engaged in PA [47].

Conversely, Bond et al. found that women with obesity and migraines spent approximately 1.5 fewer hours per day performing PA compared with controls. Intriguingly, this reduced PA was not linked to the clinical characteristics of their migraines. This study accentuates the imperative to explore the barriers to PA in these women [48]. The migraine–body composition relationship extends beyond mere PA levels. A systematic review and meta-analysis identified a potential link between migraine and obesity, which may be influenced by gender and the frequency of migraine episodes [49]. Another meta-analysis highlighted that migraine risk is elevated in both individuals with obesity and in those below the normative weight [50].

## 3. Migraines, Kinesiophobia, and Physical Activity Avoidance Behavior

Kinesiophobia, defined as “an exaggerated, irrational, and debilitating fear of physical movement and activity stemming from a sense of vulnerability to painful or new injuries” [51,52] has been associated with functional disability and pain intensity [53,54]. Some authors have speculated that kinesiophobia impacts approximately 51% to 72% of individuals suffering from chronic pain [55,56,57].

Regarding prevalence, Benatto et al. (2019) reported kinesiophobia’s presence in approximately half of those with migraines, correlating with cutaneous allodynia severity. In this study, patients with migraine often believed that PA might not alleviate their pain, and those with kinesiophobia assumed exercise might be detrimental [58].

In relation to migraine symptoms, Altay and Celenay (2023) probed the relationship between cutaneous allodynia and kinesiophobia in individuals diagnosed with migraines. Their findings unveiled a correlation between the severity of cutaneous allodynia and kinesiophobia, gastrointestinal symptoms, and migraine-related disability [59].

Disability remains paramount in diagnostic and therapeutic strategies concerning migraines. Some observations have found that the presence of kinesiophobia is associated with higher levels of migraine-related disability, an augmented fear of falls, and dizziness-induced impairment [60].

PA avoidance behaviors are a pronounced trait amongst those with migraine. During migraine episodes, an exacerbation of pain linked to motion and a consequential avoidance of certain movements (such as head motion and forward inclination) have been identified. Notably, these manifestations were distinctively prevalent and specific in individuals with migraines, contrasting with other headache typologies [61]. Moreover, Farris et al. (2019) elucidated that anxiety sensitivity, or an apprehension regarding bodily sensations, might be correlated with deliberate PA evasion in women predisposed to migraines [38]. Such insights suggest that fear-based cognition could be pivotal in shaping the exercise and movement choices of these patients.

Extending the discourse to a broader paradigm, Ruscheweyh et al. (2019) probed the association between pain avoidance and resilience in migraine contexts. Their study unveiled a positive association between social avoidance behaviors and pain-induced disability [62]. Consequently, such behaviors might exacerbate the disability engendered by migraines, curtailing the overall quality of life in affected individuals.

## 4. Depression, Self-Efficacy, Catastrophizing, and Migraine-Related Disability

Many studies have delved into the multifaceted characteristics and elements associated with migraine, encompassing psychological facets such as pain catastrophizing, depression, and self-efficacy. Klonowski et al. (2022) highlighted that psychological factors exhibit a stronger association with the perceived level of headache-related disability rather than with pain frequency [63]. Emotional states undeniably play a pivotal role in these patients. Corroborating this, Kim et al. (2021) showed altered emotional responses to pain perception in patients with migraines, identifying pain-related anxiety as a significant influencer of headache-associated disability [64].

Elsewhere, D’Amico et al. (2015) postulated that the incorporation of psychosocial variables into assessment protocols could enhance our comprehension and management of headache-associated impairment and quality of life among individuals suffering from migraines [65].

Recent observations indicate that even in those without coexisting psychiatric conditions, particular behavioral and psychological elements correlate with migraines, notably those categorized as chronic [66]. This investigation determined that patients with chronic migraines manifest heightened tendencies toward pain catastrophizing, showcasing more pronounced cognition related to helplessness and rumination compared with episodic migraine and asymptomatic controls.

Pain catastrophizing, defined as a negative cognitive and emotional response to pain, emerges as a potent determinant in pain perception and management among those with migraines [67]. Studies, including that of Mortazavi Nasiri et al. (2017), have discerned a positive correlation of pain catastrophizing with headache-related disability. Intense pain, as elucidated by their research, mediates this relationship, suggesting that maladaptive coping strategies, such as catastrophizing, might exacerbate pain experiences in these patients [68]. Additionally, Bond et al. (2015) reported that 25% of participants with migraines and obesity exhibited clinical catastrophization, which correlated with an increased frequency of attacks and a worse pain impact [69].

Conversely, the role of self-efficacy—defined as the belief in one’s ability to manage and overcome challenges—is instrumental in the context of migraines. Yousefi Afrashteh et al. (2023) found that pain self-efficacy mediated the perception of life’s meaning, social support, spiritual well-being, and pain catastrophizing with quality of life in individuals suffering from migraines [70]. In essence, the belief in one’s capability to manage pain could influence how migraine is experienced and its impact on daily life.

From another perspective, Woldeamanuel et al. (2020) offered an intriguing insight by identifying distinct natural clusters in patients with chronic migraine. Patients with higher levels of self-efficacy and physical activity reported less migraine-related disability, depression, and other symptoms [71].

It is vital to note that various psychological variables can impact pain perception and headache-related perception. Self-efficacy emerges as a crucial factor to counter and mitigate the adverse effects of migraines, whereas pain catastrophization acts as a barrier that intensifies some migraine-related symptoms. It is imperative for physiotherapists to recognize the complexity and multidimensionality of migraines to be able to provide more effective active treatment strategies.

## 5. Behavioral Modifications, Lifestyle Changes, and Physical Activity in Migraine Management

The significance of maintaining a consistent and healthy lifestyle is a recurrent theme in migraine literature. It has been highlighted that significant disruptions in daily routines, such as skipping meals or altering sleep patterns, correlate with the onset of migraine attacks [72]. Prior research has evaluated regular lifestyle behaviors, including sleep, exercise, meal patterns, and hydration, and found them to independently influence migraine occurrences. For example, in the study developed by Merril and Gibbons (2023), sleep disorders were associated with a 130% higher risk of having migraines. If participants had sleep disorders in conjunction with mental illness, this risk heightened to 289% [73]. Patients with chronic migraines exhibited fewer consistent lifestyle behaviors compared with those with episodic migraines [74]. Along these lines, the literature accentuates the pertinence of addressing health-related behaviors in episodic migraines, given that disruptions in daily routines and fluctuations in stress, sleep, and other factors can modulate both migraine onset and severity [75].

Although pharmacotherapy plays an indispensable role in migraine treatment, various studies have underscored the significance of lifestyle interventions in managing the frequency and severity of migraines, including modifications in stress management, establishing regular sleep patterns, maintaining a balanced diet, and performing physical activity [76]. In this context, maintaining consistent health behaviors is vital. Rosenberg et al. (2018) advise that because establishing and maintaining behavioral changes is challenging, they ought to be bolstered by a multidisciplinary team, and they posit that consistent daily routines could be an important tool for enhanced migraine management [75].

Among the recommended lifestyle modifications, exercise is most prominent. Barber and Pace (2020) offered a review of the contribution of exercise in migraine prevention, elucidating its potential biological and psychological benefits in migraine management. They highlighted how exercise can attenuate inflammatory markers and how an aerobic exercise regimen can markedly diminish migraine frequency and intensity [77]. In line with this, Oliveira et al. (2017), in a randomized controlled trial, probed how aerobic exercise might correlate with changes in plasma cytokine concentrations and its association with migraine prevention. Their study found that exercise induced a decline in migraine days, anxiety levels, and plasma cytokine (interleukin-12p70) levels [78]. There were significant correlations between the post-intervention levels of interleukin-12p70 levels, migraine days and anxiety scores, with the authors proposing a specific anti-inflammatory mechanism through which regular aerobic exercise could prevent migraines [78].

Addressing exercise as a preventive agent in a cohort study, Hagan et al. (2021) pinpointed how routine moderate-to-vigorous aerobic exercise (three times a week) might correlate with a reduction in headache frequency among adults with episodic migraines. This research suggests that exercise could be beneficial, especially for those already on prophylactic medication, emphasizing the merits of a combined approach [79]. This reduction in migraine frequency and other symptoms, such as migraine intensity and duration, has been observed in different clinical trials in which moderate, continuous aerobic exercise has been evaluated against usual care alone [80,81,82]. Similarly, high-intensity aerobic exercise has also been shown to reduce these symptoms in various clinical trials [82,83,84].

Other exercise modalities, such as yoga or resistance training, have also demonstrated a possible reduction in migraine symptoms and disability [85,86,87,88,89,90,91,92], showing that different exercise options may be accounted for in managing migraines. Likewise, a recently published network meta-analysis showed that yoga and moderate and high-intensity aerobic exercise were significantly superior to pharmacological treatment alone. However, the quality of evidence was very low for all these approaches [93].

## 6. Multimodal Physiotherapy in Patients with Migraine, Based on a Biobehavioral Approach

The biobehavioral paradigm for the assessment and management of patients with pain-related disorders accentuates the interplay of psychological factors, including learning history, present emotional and cognitive states, beliefs, acquired behaviors, and coping mechanisms and skills, alongside physiological disorders that influence the perception of pain and associated disability [94]. The interaction between comorbidities, such as psychiatric or other inflammatory diseases, external stressors, such as a low socioeconomic status or night-shift jobs, and migraines, which are characterized by an altered pain-processing and cortical hyper-responsiveness, frequently results in a worsening of the symptoms and a higher disability [19]. From a therapeutic standpoint, this model equips patients with the capability to self-manage pain, thereby enhancing overall functioning [95]. Given the biobehavioral traits associated with patients with migraines, we posit this model as a prime candidate for application within the physiotherapy approach.

Physiotherapy with a biobehavioral focus is defined as a therapeutic practice that combines evidence-based interventions with a multidimensional viewpoint, embracing both biological and psychosocial factors in patient diagnosis and treatment [19]. This therapeutic modality is anchored within a framework that centralizes the patient within the care paradigm, fostering a holistic understanding of their needs and circumstances [19]. Furthermore, within this approach, therapeutic procedures and decisions are navigated and recalibrated through structured clinical reasoning processes, ensuring a coherent, efficacious, and individually tailored intervention. Notwithstanding, some barriers can be present for this model, highlighting the need for the adequate structuring of this approach, given the high variability of interventions shown in studies [19].

A multimodal physiotherapy approach grounded in a biobehavioral paradigm integrates treatments such as patient therapeutic education, therapeutic exercise, and orthopedic manual therapy, among others. Evidence for this approach has been documented in patients with chronic neck pain [96], chronic lower back pain [97], temporomandibular disorders [98], chronic tension-type headache [99], and radial nerve injuries [100].

Our therapeutic proposition for addressing patients with migraines is rooted in this biobehavioral paradigm. Although there is abundant evidence on the efficacy of various therapeutic exercise prescription modalities in patients with migraines, there is currently no scientific evidence on the effect of combining patient therapeutic education with exercise. Evidence has showcased the impact of therapeutic education on reducing the frequency, intensity, and disability of patients with migraine [101]. Additionally, prior scientific findings delineate how educational strategies influence the outcomes of therapeutic exercise [102,103,104]. Therapeutic patient education is defined as “a therapeutic modality that explicitly entails a dynamic communication and interaction system with the patient, grounded in a biobehavioral paradigm, incorporating educational or formative activities that promote learning and the acquisition of adaptive skills to bolster self-management and knowledge, facilitating shifts in beliefs, attitudes, and behaviors associated with functional loss” [105].

## 7. A New Model of Therapeutic Education in Motor Behavior

**Figure 1:**
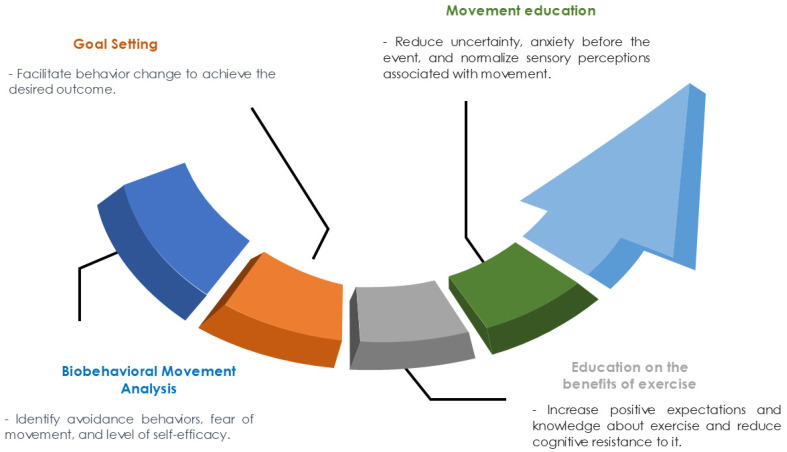
Therapeutic education on motor behavior.

**Figure 2:**
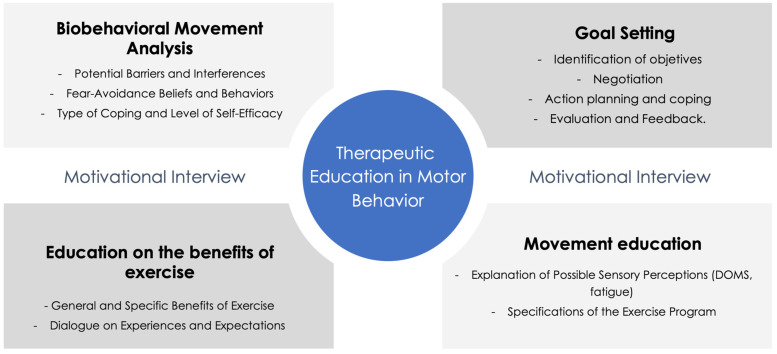
Conceptual framework for therapeutic education on motor behavior.

We propose a therapeutic education model for patients with migraines focused on increasing the effects of therapeutic exercise, which we call “therapeutic education in motor behavior”. The model establishes the four following processes or phases of action: (1) a biobehavioral analysis of movement; (2) goal setting; (3) education on the benefits of exercise; and (4) education on movement (Figure 1). The model uses motivational interviewing as a communication method for improving the motivation intrinsic to change (Figure 2).

### 7.1. Biobehavioral Movement Analysis

This phase is essential when determining the behavioral, cognitive, motivational, and emotional factors that affect the practice of therapeutic exercise. The analysis uses interviews, self-recordings, and a qualitative analysis of movement. During the analysis, it is important to identify avoidance behaviors, fear of movement, and self-efficacy levels.

### 7.2. Goal Setting

Goal setting is a collaborative process between patients and clinicians that seeks to facilitate behavioral change to achieve the desired outcome [106]. Several theories (such as the goal-setting theory, the health action process approach, and Bandura’s social learning theory) provide a rationale for the process of achieving and setting goals [107,108].

**Figure 3:**
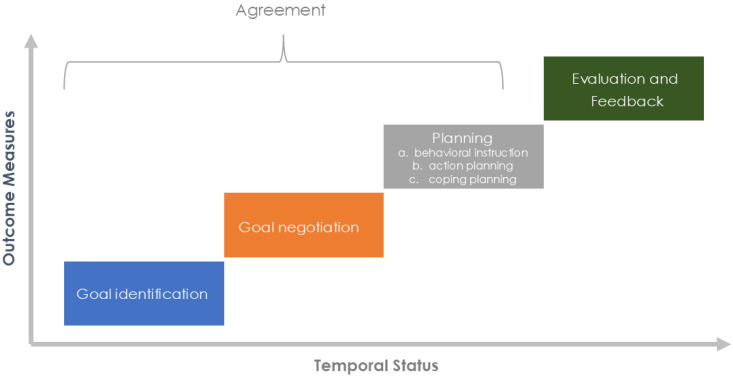
Goal-oriented therapeutic process.

In practical terms, goal setting should be developed by applying the following components: goal negotiation, goal identification, planning (action planning and coping), evaluation, and feedback [108] (Figure 3). Goal setting should also have realistic and specific goals for the patient’s condition, created with short-term goals, quantified with reliable measurements, and established based on agreement with the patients.

There are three pedagogical strategies developed during or after goal setting, detailed as follows: (1) behavioral instruction, which is based on introducing content that helps the patient incorporate and perform the exercises in their daily routine; (2) action planning, which are specific plans for achieving the objective; and (3) coping planning, in which plans are established that describe how to overcome potential barriers.

### 7.3. Education on the Benefits of Exercise

In this phase, the patient is educated on the positive effects of exercise in general and its possible effects on their signs and symptoms. The model is designed to allow patients to express their experiences and expectations regarding exercise. The aim of this phase is for the patient to increase their knowledge regarding exercise, increase their positive expectations, and reduce their cognitive resistance to exercise.

### 7.4. Movement Education

This phase includes the essential aspect that patients should know, which is the possible perceptions associated with exercise that can occur during and after the practice. This knowledge could help patients reduce their uncertainty and stress before exercising and normalize those perceptions (e.g., informing patients that the exercise can initially result in post-exercise muscle soreness, which will eventually disappear as the body physically adapts to the exercise program). It is also relevant to explain to the patients about the signs of fatigue and how to discriminate between fatigue and other symptoms.

Movement education also includes learning about the program’s specifications (exercise modalities, types of exercise, physical skills to be developed, physiological adaptations to be achieved).

## 8. Integration of the Motivational Interview in Therapeutic Education

This model of therapeutic education uses motivational interviewing as a communication technique to enhance the intrinsic motivation to change. Motivational interviewing is a highly effective communication method for physical therapy practice and is defined as a collaborative, goal-oriented, person-centered method to increase intrinsic and autonomous motivation for change [109,110], and it is a method often used as a synonym for person-centered counseling [110]. Miller and Rose (2009) have noted that motivational interviewing is relatively brief, applicable to a wide range of problem areas, complementary to other active treatment methods, and can be learned by a wide range of professionals in clinical settings [111]. Evidence has suggested that when motivational interviewing is combined with active therapy, the effect size is larger [112] and the effect is more durable [113]. Tuccero et al. (2016), suggested that motivational interviewing is effective when combined with other therapeutic approaches, such as education and cognitive–behavioral therapy [114].

Motivational interviewing focuses on the language of change and is defined by the application of communication techniques, including the use of open-ended questions, making statements, engaging in reflective listening to express empathy, using summaries, and eliciting self-motivated statements [115,116]. Motivational interviewing is structured around the following principles: expressing empathy, creating discrepancies between current behavior and future goals, managing resistance, and promoting self-efficacy [110,115]. Using empathy as a feature of patient–clinician communication reduces patient anxiety and distress and leads to improved clinical outcomes [117]. A fundamental challenge in active therapies is motivating patients to acquire adequate self-management skills, increasing patient commitment [118] and thereby improving engagement with the prescribed therapy [119].

### 8.1. Time Planning of the Model

The therapeutic education model in motor behavior is designed to be implemented over a period of 8 to 12 weeks, with weekly sessions lasting 60 to 90 min. Each week addresses a key component of the model, offering a structured yet flexible approach that adapts to the individual progress of each patient.

During the first two weeks, a biobehavioral analysis and the setting of personalized goals are conducted. This phase is crucial, as it identifies the cognitive, emotional, and behavioral factors that may influence the patient’s ability to engage in therapeutic exercise and their perception of pain. Through structured interviews, self-recordings, and qualitative movement analysis, factors such as the fear of movement, self-efficacy, and pain avoidance behaviors are explored. Based on this information, realistic and specific goals are established, which are continuously adjusted throughout the program to align with the patient’s progress. This dynamic goal-setting process ensures that interventions are tailored to the patient’s individual development.

Simultaneously, education on the benefits of exercise begins in the first week and is reinforced throughout the program. The goal of this phase is to ensure that patients understand how physical exercise can positively impact their migraine symptoms and overall well-being. By increasing their knowledge of the benefits of exercise, the aim is to reduce cognitive resistance and increase positive expectations of success.

Movement education becomes more prominent between the third and fifth weeks of the program, although it remains a central component throughout the intervention. During this phase, patients learn to interpret the sensations that may arise during and after exercise, such as fatigue, pain, or other sensory perceptions. The objective is to normalize these perceptions and help patients differentiate between normal fatigue and symptoms that may be related to migraines. This education reduces uncertainty prior to exercise and prepares patients to adopt a more proactive approach toward movement.

The therapeutic education model is complemented by the prescription of therapeutic exercise, which is tailored to each patient’s clinical evolution. This adjustment allows the exercise to be progressive and to adapt to both the patient’s physical capabilities and psychological response.

### 8.2. Human Resources Required

The successful implementation of the model requires a multidisciplinary team of specialized professionals working in a coordinated manner to address both the physical and emotional aspects of migraine treatment.

The team includes neurologists specializing in headaches, trained in motivational interviewing. These professionals are responsible for overseeing the clinical management of the patients and monitoring the progression of migraines, ensuring that the treatment is not only effective in reducing attacks but also enhances the patient’s overall well-being.

In addition, physiotherapists specializing in chronic pain and therapeutic exercise prescription play a crucial role in implementing exercise programs and educating patients about movement. These physiotherapists, also trained in motivational interviewing, guide patients in practicing safe and effective exercises, adjusting the treatment plans according to the patient’s physical and emotional responses.

Finally, clinical psychologists trained in motivational interviewing are tasked with managing the behavioral and motivational aspects of the treatment. They work to address avoidance behaviors, fear of movement, and other psychological factors that may interfere with the adoption of healthy behaviors, such as regular exercise.

It is essential that all professionals involved receive specific training in the therapeutic education model, ensuring coherent and effective program implementation while integrating both physical and psychological interventions.

### 8.3. Measures to Evaluate Model Effectiveness

Evaluating the effectiveness of the therapeutic education model is crucial to measuring its impact on both migraine symptom reduction and patient quality of life. Several clinical and behavioral measures will be employed to provide a comprehensive assessment.

The frequency and intensity of migraine attacks will be evaluated using patient-administered diaries. This will allow for a detailed follow-up of symptom progression throughout the program and will provide clear insight into the treatment’s impact on the frequency and severity of migraine episodes.

Patient quality of life will be assessed using validated scales such as the Migraine Disability Assessment (MIDAS), the 36-Item Short-Form Health Survey (SF-36), and the Headache Impact Test-6 (HIT-6). These tools provide a detailed analysis of the migraine’s impact on daily activities and the general health of patients, helping to track progress based on improved functionality and reduced disability.

Additionally, the Biobehavioral Pain and Movement Questionnaire (BioPMovQ) will be used to measure variables such as self-efficacy, movement avoidance behaviors, perceived functional capacity, and disability related to pain. This questionnaire offers an in-depth assessment of the relationships between chronic pain and motor behavior from a biobehavioral perspective, allowing for a more profound analysis of how pain influences motor behavior.

Furthermore, improvements in motor and behavioral skills will be assessed through functional tests and self-evaluations conducted by the patients. These measures will provide critical information on the patient’s functional progress and their ability to incorporate therapeutic exercises into daily life.

## 9. Exercise Prescription in Patients with Migraine

A clinical practice guideline has recently been published, and its primary aim is to offer recommendations to healthcare and exercise specialists, including neurologists, physiotherapists, and exercise physiologists, concerning prescribing exercise for patients with migraines [120]. The evidence indicates that specific exercise modalities are effective in ameliorating symptoms (e.g., headache frequency, disability, and the quality of life in patients with migraine).

Modalities with a Grade B recommendation, such as aerobic exercise [28,34,77,121,122,123,124,125,126,127,128,129,130,131,132,133,134,135,136,137,138]; continuous moderate-intensity aerobic exercise [78,79,81,139,140,141,142,143,144,145,146,147]; yoga [77,85,86,87,88,91,138,148,149,150]; and lifestyle and exercise recommendations [74,76,151,152,153,154], display promising benefits in symptom improvement. Notably, yoga, including physical postures, breathing techniques, and mindfulness exercises, is likely to decrease the frequency and disability of migraine episodes [120]. Furthermore, it is pivotal to emphasize the recommendation to integrate exercise alongside other lifestyle advices. These include maintaining consistent sleep and eating schedules, adequate hydration, stress management relaxation techniques, and abstaining from excessive medication consumption [74,76,151,152,153,154]. These interventions evidenced a reduction in pain frequency and an improvement in the quality of life of patients who suffer from episodic and chronic migraines [120]. Table 1 describes the therapeutic exercise interventions and their respective prescription parameters.

Modalities that received a Grade C recommendation, such as relaxation techniques [143,155,156,157], high-intensity interval training [83,141,142], continuous low-intensity aerobic exercise [158,159], Tai Chi [160], and resistance training [92], also exhibited benefits, albeit with a less robust evidence base compared with the Grade B-recommended modalities [120]. For instance, Tai Chi, a balance training program, demonstrated an improvement in migraine frequency in patients with episodic migraine following a 12-week intervention [160]. Relaxation techniques, including methods such as progressive muscle relaxation and visualization, have shown improvements in headache frequency after a minimum of 6 weeks of implementation [143,155,156,157].

Conversely, certain exercises, like those targeting neck endurance, showed no significant improvements in frequency, intensity, or disability in patients with episodic migraine [161]. This suggests that not all exercises are beneficial for all patients, underscoring the need for individualized prescription.

## 10. Expert Recommendations for the Prescription of Therapeutic Exercise in Patients with Migraine

Despite mounting evidence over the past decade regarding the benefits of exercise, questions persist that must be addressed to optimize the prescription of therapeutic exercise in patients with migraine. As we await empirical scientific evidence to clarify these uncertainties, the consensus of experts in exercise prescription emerges as a valuable guide for tailoring treatment for these patients. A study has recently been published on the prescription of therapeutic exercise for patients with migraine based on this consensus [162].

From the consensus findings, the optimal exercise parameters comprise sessions of 30–60 min, 3 days a week, focusing on continuous moderate-intensity aerobic exercise. Furthermore, relaxation and breathing exercises of 5–20 min daily were added [162]. Regarding additional considerations, it is recommended for initial exercise supervision to evolve toward patient self-regulation and independence. Psychological elements, including pain catastrophizing, fear-avoidance beliefs, disability, anxiety, and depression, were identified as factors that could impact adherence and exercise efficacy in these individuals. Gradual exposure to exercise might help improve these psychological factors, thereby enhancing the intervention’s efficacy [162].

On another note, experts also highlight the relevance of yoga and concurrent training as therapeutic exercise recommendations for these patients [162]. Table 2 provides details on the specific variables and characteristics of the therapeutic exercise prescription suggested by the experts.

## 11. Conclusions

The scientific literature reveals a complex relationship between migraines and physical activity. Studies indicate that individuals with migraines tend to be less physically active than asymptomatic controls, even during headache-free periods. Physical inactivity also correlates with a higher migraine prevalence. Furthermore, kinesiophobia is present in a significant percentage of patients with migraines, exacerbating functional disability and limiting quality of life. Psychological aspects such as pain catastrophizing, depression, and self-efficacy also influence migraine-related disability. These findings underscore the importance of integrating multidisciplinary therapeutic approaches, which include physical activity and behavioral intervention, for effective migraine management.

In this regard, therapeutic interventions can be approached through a biobehavioral paradigm emphasizing the interaction of psychological and physiological factors. Physiotherapy based on this approach integrates treatments such as therapeutic education and exercise, promoting pain self-management. Although evidence supports the efficacy of these interventions in other painful conditions, the combination of education and exercise in migraine warrants further research. A model of therapeutic education focused on enhancing the effects of exercise is proposed, with phases ranging from education about exercise benefits to goal setting. This model employs motivational interviewing, an effective method for enhancing intrinsic motivation for change. Recent guidelines suggest that certain exercise modalities, such as yoga and aerobic exercise, are beneficial for patients with migraines, although the necessity for individualized prescription is emphasized. Integrating exercise with other lifestyle recommendations is essential to optimize outcomes in patients with migraines.

## Figures and Tables

**Table 1:** Prescription parameters for each exercise modality included in the clinical practice guidelines.

Exercise Modality	Grade of Recommendation	Migraine Diagnosis	Type of Exercise	Distribution	Frequency and Total Duration	Time per Session	Intensity
Moderate intensity continuous aerobic training	B in favor of intervention	Episodic or chronic migraine	Supervised modalities: running, jogging, indoor cycling, or cross-training. Unsupervised modalities: Nordic walking, slow running, outdoor cycling, swimming, cycling ergometer, brisk walking, dancing, other activities.	Warm up from 5 to 15 min with walking, jogging, or easy cycling. Main training performed from 20 to 30 min. Cool down from 5 to 10 min with easy cycling, jogging, walking, or stretching.	2–3 times/week for 5–12 weeks.	Total duration of 30 to 50 min.	Warm-up gradually increased from 11 to 13 Borg. Main training performed between 13 and 16 Borg, 70% HRmax (±5 bpm), or at the intensity corresponding to participant’s ventilatory threshold. Cool down between 11 and 13 Borg.
Yoga	B in favor of intervention	Episodic migraine	Yoga: A comprehensive program initially conducted under supervision, where the first session or the first month is overseen by a professional. Afterward, the program continues at home with the aid of audio-visual materials, if available. Compliance with the routine can be monitored through weekly or bi-monthly phone check-ins, a patient-maintained diary or self-reported yoga log, and/or periodic visits with healthcare professionals	Initial phase: Includes a starting prayer, followed by breathing exercises, stretching, and relaxation techniques (such as the instant relaxation technique and quick relaxation technique). Eye-related and backward bending exercises. Second phase: Asanas, shavasana, pawanmuktasana, pranayama or pre-pranayama, neti exercise, standing–sitting and lying out screw position, kriya (Jalaneti followed by Kapalbhanti), sukshma vyayama, surya namaskar. Final phase: Shavasana or relaxation.	3–7 times/week for 6–12 weeks.	Total duration of 60–75 min.	-
Exercise and lifestyle recommendations	B in favor of intervention	Episodic and chronic migraine	Home-based exercise, stretching, light weight training, endurance training (primarily utilizing gym equipment), or any form of daily aerobic exercise that elevates the heart rate.	-	3–7 times/week for 6 weeks to more than 6 months.	Total duration of 20–60 min.	Main training performed at a moderate to submaximal intensity.
Relaxation techniques	C in favor of intervention	Episodic and chronic migraine	-	Six relaxation exercises focused on breathing and stress-management techniques, each lasting between 5 and 20 min, or Progressive Muscle Relaxation involving 16 muscle exercises, or a smartphone app offering a progressive muscle relaxation program.	1–6 times/week for 6–12 weeks.	Total duration 15 min to 120 min.	-
High-intensity aerobic interval training	C in favor of intervention	Episodic migraine	Running on a treadmill. Bicycle. Supervised.	Warm-up: 400 m of light running on a treadmill combined with two skipping exercises or 10 min of cycling. Main session: high-intensity interval training, either running on a treadmill or using a bicycle. Cool down: 400 m of light running on a treadmill followed by stretching exercises or 5 min of cycling.	2–3 times/week for 8–12 weeks.	Main training = 10–40 min. High-intensity–moderate-intensity intervals (min) = 3–4. High–moderate intensity intervals were repeated four times.	High intensity: Gradual progression over 8 weeks from a Borg scale rating of 11 to 18, or from 60% to 80% of VO2 max. The peak high-intensity level reaches 90%–95% of maximum heart rate (HRmax). The maximum intensity during active rest periods reaches 70% of HRmax.
Low-intensity aerobic exercise	C in favor of intervention	Episodic migraine	Home-based active exercise or fast walking outdoors, not supervised.	Warm-up exercises for 10 min. Main training performed for 20–40 min. Resting period performed for 10 min.	3 times/week for 6–12 weeks.	Total duration 40 min.	Main training performed at 60% HRmax.
Exercise and relaxation techniques	C in favor of intervention	Episodic and chronic migraine	Relaxation exercises combined with stationary cycling, gymnastics with music, aerobic and strength training, or a mix of stretching, isometric exercises, and walking. Supervision status was not specified.	Warm-up: 5–10 min. Main session: 30 min of moderate aerobic exercise or a combination of 15–25 min of aerobic activity with 10–20 min of strength training; alternatively, self-stretching of the neck muscles (30 s per stretch, 3 repetitions), isometric neck exercises (5 s per hold, 10 repetitions), followed by 30 min of walking. Progressive muscle relaxation: 15 min. Cool down or stretching: 5 min.	2–3 times/week for 6–12 weeks.	Total duration 45–60 min.	-
Neck strength exercise	C against the intervention	Episodic migraine	Strength exercise for superficial and deep flexor and extensor craniocervical musculature with home exercise for craniocervical musculature and stretching.	First stage: deep muscle training, two sets of 10 reps for deep flexor and extensor musculature, for 6 weeks. Progression was individualized in terms of the number of sets, repetitions, and endurance. Second phase: focused on training both deep and superficial muscles over the following 2 weeks, with three sets of 15 repetitions targeting the superficial flexor and extensor muscles.	1 day per week under supervision and 2 times/day every day with home exercises for 8 weeks.	Total duration 20 min.	-
Tai Chi	C in favor of intervention	Episodic migraine	A modified 33-movement short form of Yang-style Tai Chi Chuan, including the ‘closing’ form. The protocol consisted of both supervised and unsupervised practice.	Warm-up: 10 min of stretching. Main session: 45 min, where participants focus on learning individual Tai Chi movements during the first 5 weeks. In weeks 6 through 12, participants complete the full Tai Chi exercise routine, performing it three times per session. Cool-down: 5 min of stretching.	5 times/week for 12 weeks.	Total duration 60 min.	-
Resistance exercise	C in favor of intervention	Episodic migraine	Resistance training exercises included the use of dumbbells, arm pull-downs, arm pull-overs, sit-ups, leg curl machines, and leg extension machines. Supervision details were not provided.	Warm-up: 15 min consisting of jogging, stretching, and weightlifting. Main session: 30 to 45 min, with two to three sets of 8 to 15 repetitions each for the exercises, including arm pull-downs, arm pull-overs, sit-ups, leg extensions, and leg curls. Cool-down: 5 min of active recovery and stretching movements.	3 times/week for 8 weeks.	Total duration 30 to 45 min.	The main training was progressively carried out, increasing from 45% to 75% of the RM.
Qi-Gong	D in favor of intervention	Episodic migraine	Supervised exercise. Ju Fu (Gentle Wind) method.	The initial in-person session introduced the history of Qi-Gong along with the exercise sequence known as Ju Fu (Gentle Wind). A Qi-Gong exercise DVD was provided, replicating the content of the first and subsequent lessons for home practice. Two additional in-person sessions were conducted to reinforce training and increase the complexity and duration of the Kiko sequence.	Daily home practice over a period of 3 months. Two additional in-person sessions were held every 30 days.	Total duration at least 10 min	-

Abbreviations: bpm, beats per minute; HR, heart rate; HRmax, maximal heart rate; m/min, meters/minute; min, minutes; RM, repetition maximum; VO2 max, maximal oxygen uptake.

**Table 2:** Prescription parameters, considerations, and recommendations suggested by the experts.

**Prescription Parameters**	
*Aerobic exercise for migraines*	From 30 to 60 min of moderate-intensity continuous aerobic exercise, 3 days per week, for at least 8 weeks to improve migraine symptoms.
	The use of the Borg scale or the Talk Test to reach an optimal intensity during aerobic exercise in patients with migraines.
	Other alternatives for migraine symptom improvement, including moderate-intensity interval aerobic training or a mix of moderate- and high-intensity aerobic exercises, amounting to over 90 min per week.
	Throughout the adaptation phase, the duration of aerobic exercise per session should gradually increase, beginning at 30 min.
*Relaxation and breathing exercises for migraines*	From 5 to 20 min of a relaxation program based on relaxation, breathing and stress-management techniques practiced every day to improve migraine symptoms.
**Considerations**	
*Aerobic exercise*	Adapt aerobic exercise intensity depending on patients’ experiences and worries about exercise, fitness level, and migraine phase.
	An exercise intervention initially supervised during the early stages of migraine treatment, followed by a gradual transition to unsupervised exercise once patients are capable of self-regulating their exercise routine, understanding the proper exercise dosage, and managing their perceived exertion levels.
*Psychological factors*	The detrimental effects of magnification, learned helplessness, fear-avoidance beliefs, disability, anxiety, and depression on patients’ adherence to exercise.
	The detrimental impact of magnification, learned helplessness, fear-avoidance beliefs, anxiety, and depression on patients’ response to exercise.
	The positive impact of baseline physical activity and self-efficacy on patients’ adherence to exercise.
	The positive impact of self-efficacy on patients’ response to exercise.
*Exercise indications and contraindications in migraine*	If exercise evokes migraine, a gradual exposure to exercise should be implemented.
**Recommendations**	
	Yoga intervention to improve migraine symptoms.
	Concurrent exercise training (a combination of resistance exercise and aerobic exercise) to improve migraine symptoms.
